# HIV-1 attachment inhibitor prodrug BMS-663068 in antiretroviral-experienced subjects: week 24 sub-group analysis

**DOI:** 10.7448/IAS.17.4.19529

**Published:** 2014-11-02

**Authors:** Cynthia Brinson, Jacob Lalezari, Latiff H Gulam, Melanie Thompson, Juan Echevarria, Sandra Treviño-Pérez, David Stock, Joshi R Samit, Hanna J George, Max Lataillade

**Affiliations:** 1 Family Medicine, Austin Branch and Central Texas Clinical Research, Southwestern Medical School, Austin, TX, USA; 2 Quest Clinical Research, N/A, San Francisco, CA, USA; 3 Maxwell Clinic, N/A, Durban, South Africa; 4 AIDS Research Consortium of Atlanta, N/A, Atlanta, GA, USA; 5 Infectious and Tropical Medicine, Hospital Nacional Cayetano Heredia, Lima, Peru; 6 HIV Research Department, Mexico Centre for Clinical Research, Mexico City, Mexico; 7 Research and Development, Bristol-Myers Squibb, Wallingford, CT, USA; 8 Research and Development, Bristol-Myers Squibb, Princeton, NJ, USA

## Abstract

**Introduction:**

BMS-663068 is a prodrug of BMS-626529, an attachment inhibitor that binds directly to HIV-1 gp120, preventing initial viral attachment and entry into the host CD4+ T-cell. AI438011 is a Phase IIb, randomized, active-controlled trial investigating the safety, efficacy and dose–response of BMS-663068 versus atazanavir/ritonavir (ATV/r) in treatment-experienced (TE), HIV-1-positive subjects.

**Materials and Methods:**

Antiretroviral TE subjects (exposure to ≥1 antiretroviral for ≥1 week) with susceptibility to all study drugs (BMS-626529 IC_50_ 100 nM), were randomized equally to four BMS-663068 arms (400 or 800 mg, BID; 600 or 1200 mg, QD) and a control group (ATV/r 300/100 mg QD) with tenofovir disoproxil fumarate (TDF) + raltegravir (RAL). A sub-group analysis of viral efficacy and immunologic reconstitution is presented.

**Results:**

A total of 251 subjects were treated. Median age was 39 years, 60% were male and 38% were white. Median baseline (BL) viral load (VL) was 4.85 log_10_ c/mL (43%; 100,000 c/mL) and median CD4+ T-cell count was 230 cells/mm^3^ (38%; 200 CD4 cells/mm^3^). Through Week 24, response rates (HIV-1 RNA 50 c/mL) were comparable across all BMS-663068 arms and the ATV/r arm regardless of gender, age and race. Response rates for subjects with BL VL 100,000 c/mL (BMS-663068, 82-96%; ATV/r, 93%) were higher than those for subjects with BL VL ≥100,000 c/mL (BMS-663068, 70-87%; ATV/r, 73%); however, there were no substantial differences in response across the BMS-663068 and ATV/r arms in either sub-group. Response rates for subjects with BL CD4+ cell counts ≥200 cells/mm^3^ (87-96%) were higher than those for subjects with BL CD4+ cell counts 200 cells/mm^3^ (62–82%); however, no substantial differences in response were seen across the BMS-663068 and ATV/r arms in either sub-group. Mean changes in CD4+ T-cell counts from BL were similar across all arms regardless of gender, age and BL CD4+ T-cell count.

**Conclusion:**

Virologic response rates were similar across the BMS-663068 and ATV/r arms in TE subjects, regardless of BL demographic characteristics (gender, race, age), BL HIV-1 RNA, or BL CD4+ T-cell count. Mean increases in CD4+ T-cell counts across the BMS-663068 arms were consistent with ATV/r, regardless of gender, age and BL CD4+ T-cell count. These results support continued development of BMS-663068.

**Note:**

Previously submitted at IDWeek, Philadelphia, PA, 8 October 2014.

